# Relevance of Bcl-x expression in different types of endometrial tissues

**DOI:** 10.1186/1756-9966-29-14

**Published:** 2010-02-23

**Authors:** Xiaoxin Ma, Yanhui Zhao, Yanxia Li, Hongwei Lu, Yuanqi He

**Affiliations:** 1 Department of Obstetrics & Gynecology, Shengjing Hospital of China Medical University, Shenyang(110004), China; 2 China Medical University, Shenyang(110004), China

## Abstract

**Objectives:**

To explore the roles of Bcl-xl and Bcl-xs in the development and progression of endometrial carcinoma, and to analyze the correlation between Bcl-xl and Bcl-xs.

**Methods:**

RT-PCR and Western-blot assay were applied to detect the expressions of Bcl-xl and Bcl-xs in endometrial tissues of various histomorphologic types.

**Results:**

The Bcl-xl expression levels of simple and atypical hyperplasia endometrial tissues were not significantly different from that of normal endometrial tissue (both *P *> 0.05). On contrary, Bcl-xl expression in endometrial carcinoma tissue was significantly higher than the normal endometrial tissue (*P *= 0.00), which was correlated with the pathological grading of endometrial carcinoma (F = 5.33, *P *= 0.02). In addition, Bcl-xs mRNA level in simple hyperplasia endometrial tissue had no significant difference compared to that in normal endometrial tissue (*P *= 0.12), while the levels of atypical hyperplasia and endometrial carcinoma endometrial tissues were significantly different from the normal endometrial tissue (both *P *= 0.00). Furthermore, level of Bcl-xs mRNA was correlated with the clinical staging and lymph node metastasis of the endometrial carcinoma (*P *< 0.05). The expressions of Bcl-xl and Bcl-xs were negatively correlated with each other (*r *= -0.76).

**Conclusion:**

The abnormal expressions of Bcl-xs and Bcl-xl were one of the molecular mechanisms for the pathogenesis of endometrial carcinoma, and altered ratio between these two might involve in the onset of endometrial carcinoma.

## Introduction

Endometrial carcinoma is one of the common malignant tumors of female genital tract. The incidence of endometrial carcinoma continued to increase annually and it has replaced cervical cancer in some countries as the most common malignant tumors of female genital tract[1]. However, the molecular biological mechanisms involved in the pathogenesis of endometrial carcinoma remain unclear. Recent studies find that Bcl-2 family is a major tumor suppressor gene family in association to the pathogenesis of endometrial carcinoma. As a regulatory point for caspase activation and mitochondria function, Bcl-2 gene family functions as a common pathway for transmission of cell apoptosis signals to regulate cell survival and apoptosis[2]. There are at least 15 members in the Bcl-2 family[3,4], among which Bcl-2 and Bcl-x are major genes involving in the development and progression of tumors and therefore attract much attentions. Bcl-xl and Bcl-xs are encoded by Bcl-x gene, where the abnormal expression of such in various tumors including breast cancer, multiple myeloma and thyroid cancer etc. has been reported in many domestic and foreign literatures[5-7]. However, few report has shown the levels of Bcl-xl and Bcl-xs in endometrial carcinoma tissue. The objective of this study was to investigate the roles of Bcl-xl and Bcl-xs in the development and progression of endometrial carcinoma.

## Materials and methods

### Material

Experimental group included endometrial tissues from 50 patients, who underwent surgery or hysteroscopy for suspected endometrial lesions in the Department of Obstetrics and Gynecology department in Shengjing Hospital of China Medical University from December 2005 to October 2006, including 6 cases of simple hyperplasia, 12 cases of atypical hyperplasia and 32 cases of endometrial carcinoma. Tissues with endometrial lesions were extracted for subsequent experiments. Control group included normal endometrial tissues from patients who underwent hysterectomy for carcinoma of the cervix, including tissues in proliferative phase(6 cases) and tissues in secretory phase(4 cases), total of 10 cases. Patients in experimental group aged 34 ~70 years old with an average age of 52 ± 5.04 years old, while the range of ages in control group was 37 ~59 years old with an average age of 48 ± 2.13 years old. Patients did not receive radiotherapy, chemotherapy or hormone therapy before the surgery and all cases were confirmed by histopathology. 32 cases of endometrial carcinoma were graded for surgical and pathologic stages according to the criteria in FIGO 1988: 22 cases of stage I, 4 cases of stage II and 6 cases of stage III endometrial carcinoma. Histological grading: 14 cases of G1, 12 cases of G2 and 6 cases of G3 for endometrial carcinoma. Myometrial invasion classification: 10 cases in stage Ia, 16 cases in stage Ib and 6 cases in stage Ic. Patients were also grouped according to the status of lymph node metastasis: 6 cases with lymph node metastasis and 26 cases free of lymph node metastasis.

### Methods

#### RT-PCR technique to detect the expressions of Bcl-xl and Bcl-xs mRNA

Total tissue RNA was extracted by following protocol provided in the TRIzol reagent kit (DaLian TAKARA Biotechnology Company). The 1^st ^strand of cDNA was synthesized according to protocol provided in the Reverse Transcription kit (Shanghai Invitrogen Biotechnology Co. Ltd.), while using a total of 15 μl of reaction system with 1.5 μl template RNA. The cDNA product was stored at -20°C for experiments. β-actin was included as an internal control and PCR assay was performed to amplify target genes. The volume of PCR reaction system was 25 μl: 3 μl template cDNA, 2.5 μl 10 × buffer, 2 μl 2.5 mM dNTP, 0.1 μl of each primers, and 0.2 μl 5 u/μl Taq-E and the total reaction volume was raised to 25 μl using deionized water. Bcl-xl primer sequences were: upstream 5'-GGCAACCCATCCTGGCACCT-3', downstream 5'-AGCGTTCCTGGCCCTTTCG-3', yielding predicted amplification product of 472 bp. Bcl-xs primer sequences were: upstream 5'-GAGGGAGGCAGGCGACGAGTTT-3', downstream 5'-ATGGCGGCTGGACGGAGGAT-3', yielding predicted amplification product of 216 bp. β-actin primer sequences were: upstream 5'-GTGGGGCGCCCCAGGCACCA-3, downstream 5'-CTCCTTAATGTCACGCACGATTTC-3', yielding predicted amplification product of 498 bp. β-actin was used as internal control to normalize different reactions. PCR reaction was performed on an thermocycler (PTC-100™, USA). Amplification conditions for Bcl-xl were: initial denaturation at 94°C for 3 min, then proceeding with the following reaction conditions: a total of 35 cycles of denaturation at 94°C for 45 s, annealing at 59°C for 45 s, and extension at 72°C for 60 s before final extension at 72°C for 7 min. As for Bcl-xs, the process included: initial denaturation at 94°C for 3 min, then proceeding with the following reaction conditions: a total of 35 cycles of denaturation at 94°C for 40 s, annealing at 60°C for 60 s, and extension at 72°C for 60 s, before final extension at 72°C for 7 min. 5 μl PCR product was subjected to 2% agarose gel electrophoresis (150 v) for 60 min and stained with ethidium bromide. RT-PCR amplification product was then observed under UV light. ΦX174Hinc II (TAKARA Co.) was included as the standard for relative molecular size. 1D KodaK image analysis software was used to observe and capture images. Optical density (*A*) ratio of target gene and β-actin RT-PCR amplification products was calculated to determine the relative mRNA content of the target gene.

#### Western-blot assay to determine the expressions of Bcl-xl and Bcl-xs/l protein

Cytosolic protein was extracted and sample OD values were determined by phenol reagent assay (0.305~1.254). All samples were adjusted to equal concentration and sampling buffer was added. Total of 40 ug protein was loaded onto 10% polyacrylamide gel for 2 h electrophoresis and β-actin was used as loading control. After electric transferring, membrane was washed with TBS once, blocked by TBS containing 5% (v/v) skim milk overnight and then washed with TTBS for 3 times, 5 min for each wash. Mouse anti-human Bcl-xl monoclonal antibody and mouse anti-human Bcl-xs/l monoclonal antibody were added (1:500 dilution for both antibodies in TTBS containing 1% BSA), before 2 hours of incubation at room temperature. Next, membranes were washed by TTBS for 3 times and horseradish peroxidase-labeled mouse anti-rabbit IgG secondary antibody was added (1:500 dilution), The whole setup was incubated at room temperature for 1 h and washed by TTBS for 3 times, 5 min for each and finally washed by TBS for 5 min. An automatic electrophoresis gel image analysis system (Chemi Imageer 5500) was used to analyze optical intensities of the protein bands. The equation of relative optical density (*A*) = optical density of the target protein/optical density of actin, was used to perform semi-quantitative analysis.

#### Statistical analysis

SPSS13.0 statistical software was used to perform unpaired *t*-test, one-way ANOVA and correlation analysis. *P *< 0.05 was set as the criteria for statistical significance.

## Results

### Expressions of Bcl-xl and Bcl-xs mRNA in different types of endometrial tissues

**Figure 1:**
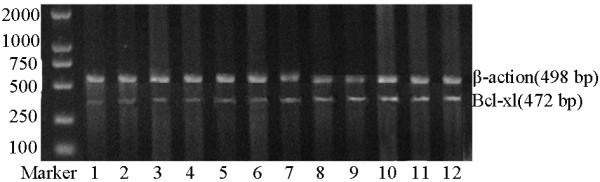
**Bcl-xl mRNA(RT-PCR)**. 1, 2: Normal endometrium; 3, 4: Simple hyperplasia endometrial tissue, 5, 6: Atypical hyperplasia endometrial tissue; 7~12: Endometrial carcinoma tissue.

RT-PCR result showed that tissues of expressed Bcl-xl mRNA in order from low to high levels Bcl-xl mRNA expressions were normal endometrium, simple hyperplasia endometrial tissue, atypical hyperplasia endometrial tissue, and endometrial carcinoma tissue (Fig. 1). Although level of Bcl-xl mRNA was slightly unregulated in simple hyperplasia endometrial tissue, it was not significantly different than that of normal endometrial tissue (*t *= -1.51, *P *> 0.05). In addition, no significant difference was detected between Bcl-xl mRNA level of atypical hyperplasia endometrial tissue and that of normal endometrium (*t *= 0.90, *P *> 0.05). On contrary, Bcl-xl expression in endometrial carcinoma tissue was significantly higher than in normal endometrial tissue (*t *= 15.44, *P *< 0.05). Expression of Bcl-xl mRNA was not correlated with clinical staging, myometrial invasion and lymph node metastasis of the endometrial carcinoma, but correlated with histological grade (F = 5.33, *P *= 0.02) (Table 1).

**Table 1:** Contents of Bcl-xl and Bcl-xs mRNA in different types of endometrial tissue and correlation with pathological parameters of the endometrial carcinoma

Classification	Bcl-xl mRNA expression		Bcl-xs mRNA expression	
	
	χ ± S	*P *value	χ ± S	*P *value
Normal endometrium	0.35 ± 4.37		0.93 ± 3.05	

Simple hyperplasia	0.38 ± 3.25	0.13	0.89 ± 2.00	0.12

Atypical hyperplasia	0.37 ± 3.93	0.38	0.68 ± 0.10	0.00

Endometrial carcinoma	0.75 ± 0.13	0.00	0.49 ± 0.14	0.00

Degree of Pathological Differentiation				

Well-differentiated	0.85 ± 7.23		0.52 ± 0.14	

Moderately-differentiated	0.70 ± 7.60	*F *= 5.33	0.45 ± 0.16	*F *= 0.40

Poorly-differentiated	0.70 ± 1.44	*P *= 0.02	0.48 ± 7.57	*P *= 0.68

Clinical Staging				

Stage I	0.74 ± 0.15		0.55 ± 7.67	

Stage II	0.79 ± 0.10	*F *= 0.57	0.41 ± 2.83	*F *= 30.87

Stage III	0.82 ± 0.15	*P *= 0.58	0.21 ± 7.77	*P *= 0.00

Lymph Node Metastasis				

No	0.82 ± 0.16	*F *= 2.31	0.51 ± 9.16	*F *= 0.64

Yes	0.79 ± 0.10	*P *= 0.73	0.25 ± 6.70	*P *= 0.00

Depth of Myometrial Invasion				

0	0.82 ± 7.26		0.58 ± 7.07	

≤ 1/2	0.76 ± 0.11	*F *= 3.22	0.45 ± 0.16	*F *= 1.73

> 1/2	0.64 ± 4.73	*P *= 0.07	0.45 ± 6.03	*P *= 0.22

**Figure 2:**
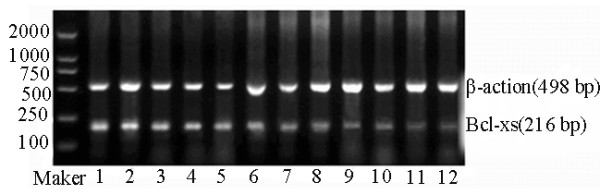
**Bcl-xs mRNA(RT-PCR)**. 1, 2: Normal endometrium; 3, 4: Simple hyperplasia endometrial tissue, 5, 6: Atypical hyperplasia endometrial tissue; 7~12: Endometrial carcinoma tissue.

Furthermore, tissues of expressed Bcl-xl mRNA in order from low to high levels Bcl-xs mRNA levels were normal endometrium, simple hyperplasia endometrial tissue, atypical hyperplasia endometrial tissue and endometrial carcinoma tissue (Fig. 2). Although its expression was slightly elevated in simple hyperplasia endometrial tissue, no significant difference was detected compared to normal endometrial tissue (*t *= 1.80, *P *> 0.05). On contrary, its expression was significantly different between atypical hyperplasia endometrial tissue and normal endometrium (*t *= 5.17, *P *< 0.05). In addition, Bcl-xs expression in endometrial carcinoma tissue was significantly higher than that in normal endometrium (*t *= 6.88, *P *< 0.05) (Table 1). Expression level of Bcl-xs mRNA was correlated with clinical staging and lymph node metastasis of the endometrial carcinoma, but not related to myometrial invasion and pathological staging.

### Expressions of Bcl-xl and Bcl-xs/l protein in different types of endometrial tissues

**Figure 3:**
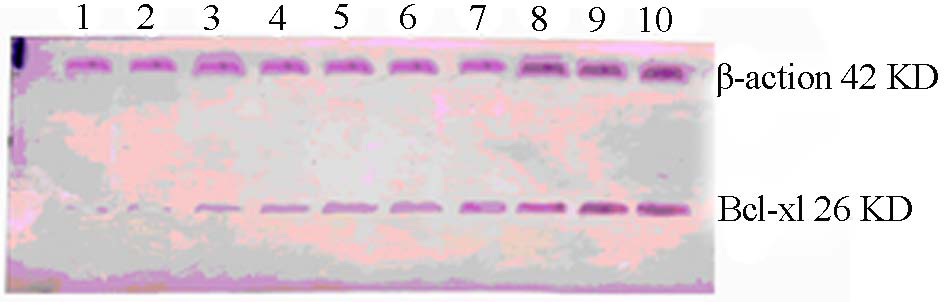
**Expression of Bcl-xl protein in different types of endometrial tissues**. 1, 2: Normal endometrium; 3, 4: Simple hyperplasia endometrial tissue, 5~7: Atypical hyperplasia endometrial tissue; 8~10: Endometrial carcinoma tissue.

**Figure 4:**
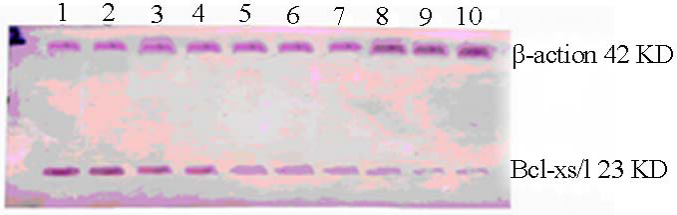
**Expression of Bcl-xs/l protein in different types of endometrial tissue**. 1, 2: Normal endometrium; 3, 4: Simple hyperplasia endometrial tissue, 5~7: Atypical hyperplasia endometrial tissue; 8~10: Endometrial carcinoma tissue.

Immunoblotting results showed that Bcl-xl protein expression had matched pattern with expression of Bcl-xl mRNA in different types of endometrial tissues, For example, these two were positively correlated (*r *= 0.44, *P *= 0.015). In other words, expressions of these two proteins were relatively low in normal endometrial tissue, while elevated expression could be detected in both simple hyperplasia and atypical hyperplasia endometrial tissues (Fig. 3). In addition, expressions of Bcl-xl and Bcl-xs/l proteins did not show a significant difference between simple hyperplasia and normal endometrial tissues (*t *= -0.61, *P *> 0.05) and the expression in atypical hyperplasia endometrial tissue was not significantly different from that in normal endometrial tissue (*t *= -0.61, *P *> 0.05). Expressions of Bcl-xl and Bcl-xs/l proteins were further upregulated in endometrial carcinoma tissue to a level significantly different from that of normal endometrial tissue (*t *= -2.22, *P *= 0.04). Furthermore, expression of Bcl-xl protein correlated with pathological staging of the tissue sample (see Table 2). Trend of Bcl-xs/l protein expressions in different types of endometrial tissues matched that of Bcl-xs mRNA expression. Specifically, no significant difference was found in Bcl-xs/l protein between simple hyperplasia and normal endometrial tissues (*t *= 0.33, *P *= 0.75). However, significant differences of Bcl-xs/l expression were detected between normal endometrial tissue and atypical hyperplasia endometrial tissue (*t *= 2.42, *P *= 0.04), as well as between normal endometrial tissue and endometrial carcinoma tissue (*t *= 4.14, *P *= 0.00) (Fig. 4). Expression of Bcl-xs/l protein did not correlated with degree of myometrial invasion and pathological staging, but significantly correlated with clinical staging and lymph node metastasis of the sample (see Table 2).

**Table 2:** Contents of Bcl-xl and Bcl-xs/l protein in different types of endometrial tissue and correlation with pathological parameters of the endometrial carcinoma

Classification	Bcl-xl protein expression		Bcl-xs/l protein expression	
	
	χ ± S	*P *value	χ ± S	*P *value
Normal endometrium	41.00 ± 21.05		105.60 ± 33.05	

Simple hyperplasia	49.00 ± 11.36	0.57	96.00 ± 50.48	0.75

Atypical hyperplasia	49.00 ± 11.36	0.56	73.00 ± 4.47	0.04

Endometrial carcinoma	90.88 ± 48.33	0.04	54.50 ± 18.49	0.00

Degree of Pathological Differentiation				

Well-differentiated	109.29 ± 39.06		57.71 ± 22.33	

Moderately-differentiated	71.50 ± 13.53	*F *= 4.65	56.50 ± 17.81	*F *= 0.32

Poorly-differentiated	56.67 ± 17.21	*P *= 0.03	46.67 ± 4.04	*P *= 0.74

Clinical Staging				

Stage I	85.17 ± 50.83		61.17 ± 16.03	

Stage II	108.00 ± 48.08	*F *= 0.30	45.50 ± 2.12	*F *= 4.02

Stage III	108.00 ± 52.33	*P *= 0.74	30.50 ± 6.36	*P *= 0.04

Lymph Node Metastasis				

No	88.43 ± 49.33	*F *= 0.06	55.43 ± 21.58	*F *= 0.95

Yes	108.00 ± 52.33	*P *= 0.61	30.00 ± 5.66	*P *= 0.02

Depth of Myometrial Invasion				

0	76.80 ± 18.78		65.60 ± 19.92	

≤ 1/2	86.00 ± 38.58	*F *= 1.13	52.25 ± 18.55	*F *= 1.34

> 1/2	127.33 ± 94.99	*P *= 0.35	46.67 ± 2.52	*P *= 0.30

### Correlation analysis between Bcl-xl and Bcl-xs

Correlation analysis identified a negative correlation between Bcl-xl gene and Bcl-xs gene in different types of endometrial tissues (r = -0.76, P = 0.00). Bcl-xl protein was negatively correlated with expression of Bcl-xs/l protein (r = -0.39, *P *= 0.04) and Bcl-xs gene was positively correlated with Bcl-xs/l protein expression (r = 0.73, *P *= 0.00).

## Discussion

As a member of Bcl-2 gene family, role of Bcl-x in the development and progression of tumors has received more and more attentions. Bcl-x gene was cloned by Boise[8] in 1993 by screening a chicken lymphocyte cDNA library using mouse Bcl-2 cDNA as the probe. Bcl-x has dual regulatory roles after activation. It is localized at 20q11.21 and a different splicing site at the 5' terminus of its 1^st ^mRNA exon leads to two fragments: a longer fragment Bcl-xl and a shorter fragment Bcl-xs. In recent years, expression of Bcl-x gene products (Bcl-xl and Bcl-xs) in some tumors has been reported in domestic and foreign studies. However, the expression status in endometrial carcinoma tissue has rarely been characterized yet.

### Expression of Bcl-xl in endometrial carcinoma tissue and the significances

Bcl-xl contains 241 amino acids and BH1-BH4 4 homologous sequences. Its sequence is 43% identical to that of Bcl-2 and their functions are similar too. Bcl-xl could inhibit cell apoptosis through forming heterodimer with Bax in cytosol. Studies found that Bcl-xl could inhibit apoptosis in a Bcl-2-independent manner. It could inhibit cell apoptosis mediated by many apoptosis-inducing factors, which was far upstream in regulation of apoptosis. Bcl-xl protein was highly expressed in some tumors with low level of Bcl-2. Some researchers believed that Bcl-xl protein might have substituted the function of Bcl-2 in some tumors. Under certain condition, this protein has stronger apoptosis-inhibitory effect over Bcl-2, indicating the key role of Bcl-xl in the process of cell transformation.

Studies showed that tumor cell apoptosis could be induced by lowering the Bcl-xl expression in human prostate cancer tissue[9]. Furthermore, researches demonstrated that induction of tumor cell apoptosis could be achieved through inhibiting the expression of Bcl-xl in malignant pleural mesothelioma[10]. Boehmdenf et al. [11]also showed that Bcl-xl expression in head and neck squamous cell carcinoma was significantly different among different types of pathological grading, while the expression of Bcl-xl protein in human prostate cancer specimens was closely correlated with the Gleason scoring and metastasis of human prostate cancers[12]. Therefore, Bcl-xl plays an important role in pathogenesis of tumor as an anti-apoptotic factor, and chemotherapy-resistance of the tumor cell may be associated with high level of Bcl-xl expression [13,14].

Our study found that expressions of Bcl-xl mRNA and protein were slightly increased in simple hyperplasia and atypical hyperplasia endometrial tissues, while significantly increased in endometrial carcinoma tissue. In addition, Bcl-xl expression was correlated with the pathological grading of endometrial carcinoma, suggesting that elevation in Bcl-xl disrupted the regulation of signal transduction and normal gene expression, while it led to abnormal endometrial cell proliferation differentiation and eventually endometrial carcinoma. Therefore, we concluded that apoptotic inhibition caused by abnormal Bcl-xl expression might be one of the pathogenetic mechanisms of endometrial carcinoma, and this abnormal expression might also be associated with the malignant behaviors of endometrial carcinoma.

### Expressions of Bcl-xs mRNA and Bcl-xs/l protein in endometrial carcinoma and the significances

Bcl-xs has 63 amino acids less than Bcl-xl (BH1 and BH2 region). Its function is similar to that of Bax, which is to inhibit Bcl-2 activity and promote cell apoptosis[4]. Sumantran et al. [5] used adenoviruses as vector to introduce Bcl-xs into breast cancer cell line. Their results showed that adv-Bcl-xs transfection could induce tumor cell apoptosis. In 1996, Ealovega et al. [15]constructed a replication-deficient adenovirus as vector to transiently express Bcl-xs in MCF-7 human breast cancer cell line and nude mice breast cancer tissues. They found that Bcl-xs overexpression could induce apoptosis of MCF-7 cells. Further studies have shown that adv-Bcl-xs could infect breast cancer cells *in vitro *or *in vivo *to induce growth inhibition and death of breast cancer cells. This inhibitory and pro-apoptotic effects were more prominent with increased virus titer and increased Bcl-xs gene copies carried by the virus[16]. Our results showed that expressions of Bcl-xs mRNA and Bcl-xs/l protein slightly decreased in normal and simple hyperplasia endometrial tissues, while significantly decreased in atypical hyperplasia and endometrial carcinoma tissues, suggesting that abnormal expressions of these two played important roles in the early stage of endometrial carcinoma development. It was possible that low-expression of Bcl-xs led to inhibition of apoptosis, and thus abnormal endometrial cells threatening the body function could not be eliminated, resulting in endometrial carcinoma.

### The correlation between expressions of Bcl-xl and Bcl-xs in different types of endometrial tissues

Bcl-xs can form heterodimer with Bcl-xl. Ratio of these two affects the sensitivity and resistance of cells to variety of apoptotic factors and determines the activity of caspases, which are the final pathway for apoptosis in many different cells. Many Bcl-2 gene family members form a system with other members to modulate apoptosis, especially Bcl-2, Bcl-xs and Bax. Qiang Wang et al. [17] used in situ hybridization to test the expression statuses of Bcl-xl and Bcl-xs in post-ischemic brain tissue undergoing mild hypothermia treatment. They confirmed that ratio between Bcl-xl and Bcl-xs concentrations determined whether apoptosis would occur or not. The expression of Bcl-xl and Bcl-xsm in different types of endometrial tissues were negatively correlated. We speculate that it might be Bcl-xs not Bcl-xl expression that is dominant in normal endometrial tissue. With progression of endometrial lesion, Bcl-xl expression increased while Bcl-xs expression decreased gradually. When Bcl-xl expression becomes dominant, endometrial carcinoma will be induced. The ratio between these two has certain impact on the development of endometrial cancer.

Since no specific Bcl-xs antibody is available now, we could not detect its expression directly using Western blot. In our study, we used Bcl-xs/l antibody that recognized a common motif of Bcl-xl and Bcl-xs, and primarily the motif in Bcl-xs. Our result suggested that expression of Bcl-xs/l was low in endometrial lesion tissue of high Bcl-xl expression, implying low expression of Bcl-xs in these tissues.

In summary, our results suggested that abnormal elevation of Bcl-xl expression and abnormal decrease of Bcl-xs expression played an important role in the development of endometrial carcinoma. When malignant biological behaviors of endometrial carcinoma developded, Bcl-xs gene expression was significantly decreased, providing a new tumor marker for the early diagnosis of endometrial carcinoma. Further studies on the action mechanisms of Bcl-xl and Bcl-xs gene should provide new molecular targets for gene therapy of endometrial carcinoma.

## Competing interests

The authors declare that they have no competing interests.

## Authors' contributions

XM designed the study and carried out RT-PCR technique and the Western-blot assay. YZ participated in RT-PCR technique and drafted the manuscript. YL participated in the Western-blot assay. HL participated in its design and coordination. YH participated in the manuscript drafting and performed the statistical analysis. All authors read and approved the final manuscript.
